# Efficient resin production using stimulant pastes in *Pinus elliottii* × *P. caribaea* families

**DOI:** 10.1038/s41598-022-17329-2

**Published:** 2022-07-30

**Authors:** Yang Liu, Zhe Wang, Fencheng Zhao, Ming Zeng, Fuming Li, Lifang Chen, Huishan Wu, Xiaoliang Che, Yiliang Li, Leping Deng, Suiying Zhong, Wenbing Guo

**Affiliations:** 1 Guangdong Academy of Forestry, Guangzhou, 510520 Guangdong China; 2 Guangdong Provincial Key Laboratory of Silviculture, Protection and Utilization, Guangdong Academy of Forestry, Guangzhou, 510520 Guangdong China; 3 Taishan Hongling Seed Orchard, Taishan, 529200 Guangdong China

**Keywords:** Plant sciences, Secondary metabolism

## Abstract

To address the increasing labor cost of resin tapping, more efficient methods for resin tapping need to be developed. This study aimed to evaluate the features of resinosis as affected by stimulant pastes in *Pinus elliottii* × *P. caribaea*, which is also one of the predominant resin-producing species hybrids in South China. The resin yields and resin compositions were assessed in 33 *P. elliottii* × *P. caribaea* F_1_ families, with the application of four kinds of chemical stimulants, potassium (K_2_SO_4_) paste, naphthalene acetic acid (NAA) paste, benzoic acid (BA) paste and 2-chloroethylphosphonic acid (CEPA) paste. Our results showed that all four pastes significantly increased the resin yield by at least 20% at each tapping, and 3- to fivefold increases were detected at the beginning of each year. The correlations between resin yield and growth at each tapping ranged from uncorrelated to moderately positively correlated, indicating that resin yield was mostly but not always determined by tree size. The concentration of each resin component did not change with the stimulant applications. In *P. elliottii* × *P. caribaea*, selecting a larger tree diameter at breast height and employing the chemical stimulants at the first several tapping rounds are efficient tapping procedures. Moreover, the K_2_SO_4_-based stimulant can be recommended considering its promoting effects on resin yield and the low cost of the chemicals required to produce it.

## Introduction

Pine resin, which can be converted into rosin, turpentine and their derivatives, is an important nontimber forest product^1,2^. Resin derivatives have been widely used by the chemical industry in the production of solvents, paint, ink, adhesives, cleaners, insecticides, pharmaceuticals, cosmetics, and food additives^3,4^. In China, resin tapping usually requires tapping every 2–3 days. The pine forests in China are usually steep mountains. The area that each worker can harvest is limited. Resin tapping is a drudgery and widely used methods are inefficient^5^. The high labor cost and the low price of resin are two major challenges of the resin industry. Therefore, it is necessary to reduce the tapping frequency and increase the efficiency of tapping^6,7^.

Resin extrudes from resin ducts when the pine trees are wounded^8^. Resin yield in pines is influenced by a number of internal and external factors. External factors include tapping season, climate, wound damage, burning, biotic and abiotic stress, fertilization and metabolic pathway regulators^7–11^. Chemical stimulants, which can extend wound damage, mimic pathogen signaling, or promote terpene synthesis, have been adopted to increase tapping efficiency^12^. The routinely used commercial resin stimulants are pastes containing sulfuric acid and 2-chloroethylphosphonic acid (CEPA). CEPA is an ethylene precursor. It also functions as a signaling molecule to elicit a defense response and induce resin flow. Sulfuric acid can prolong the resin flow period by maximizing the effect of wounding at the injury zone^13^. It was reported that the combination of 25% sulfuric acid plus 5% CEPA can increase the resin yield up to 36% in slash pine^14^. Furthermore, the effect of stimulant on resin yield of slash pine followed a clear seasonal pattern^15^. The combination of 20% sulfuric acid plus 4.5% CEPA increased the resin yield by 31% and 45% in spring and summer, respectively, although it had a limited effect in fall and winter. In addition to CEPA, several other chemicals can improve resin yield^12^. One of these chemicals is a pathogen signaling molecule, including salicylic acid and its precursor benzoic acid (BA). Auxin is another molecule that can promote ethylene biosynthesis and resin canal differentiation. Metal cofactors of terpene synthases, such as iron or potassium ions, are also used as replacements for CEPA^16^. It is known that either auxin (2,4-dichlorophenoxyacetic acid, 2,4-D) or salicylic acid have a similar effect to CEPA on increasing resin production^15^. In addition, it has been reported that the application of potassium (K) could lead to a higher resin yield than CEPA^9,16^. A systematic study compared the effect of auxin (naphthaleneacetic acid, NAA), BA, K and CEPA on resin yield^12^. The results showed that trees treated with CEPA produced higher amounts of resin than those treated with the other three individual chemicals. However, the results were not repeatable across years^12^. In addition, unlike NAA treatment, which could increase the concentration of β-pinene, CEPA or K treatments did not affect resin composition^12^.

The internal factors affecting resin yield mainly include genotype, tree size and anatomical structures^4,17–19^. *Pinus massoniana* and *P. elliottii* are the two major species used for resin production in Guangdong Province, China. The resin yield of *P. massoniana* is lower than that of *P. elliottii*, while the resin price is higher than that of *P. elliottii* due to their differences in resin composition^20^. For both species, the resin yield and resin composition were significantly affected by family effects. Furthermore, significant phenotypic and genetic correlations were observed between growth and resin yield^20,21^.

The first successful interspecific hybridization between *P. elliottii* and *P. caribaea* var. *hondurensis* (PCH) was undertaken in Australia in 1955. The hybrid had superior growth and a straight trunk^22,23^, which made it an ideal source for structural timber and plywood products where *P. elliottii* was traditionally planted (e.g., Australia, South America and South Africa). In China, interspecies hybridization between *P. elliottii* and *P. caribaea* has been carried out independently in Guangdong since 1992. In addition to wood utilization, it has increasingly become one of the main species for resin tapping in southern China^24^. To deploy the improved *P. elliottii* × *P. caribaea* families for resin tapping, it is necessary to understand the external and internal factors that affected resin yield in this species hybrid. Thus, in the current study, we assessed the effects of different resin stimulants on resin yield and resin composition, as well as their interaction effect with genotype and tree size.

## Results

### Treatment effect rather than family effect affecting the resin yield

There were 4 treatment groups and one control group in this study. All the trees in treatment groups tapped with applying stimulants at each tapping rounds except two rounds of free of stimulants control. At the 4th tapping round in 2018 and the first tapping round in 2019, the trees were tapped without applying stimulants to measure the family effect when free of stimulants. The data revealed that the application of stimulating paste significantly affected the resin yield at almost every tapping round in 2018 and 2019 (Table 1), except for the 3rd tapping round in 2018 (*P* = 0.0758). Inconsistent with previous studies, the family effects were not significant on the resin yield at almost every tapping round. This may largely be due to crown damage caused by a typhoon that hit the experimental area during that period. Significant treatment effects and nonsignificant family effects were detected at the 4th tapping round in 2018, indicating the residue effect of chemical stimulants. Furthermore, it was only at the first tapping round in 2019, which was free of stimulants, that family differences were resolved. This implied that the external effects when applying stimulants might be too strong to detect variation among families.

**Table 1:** Significance levels (*P *values) from the analysis of variance conducted on the weekly harvest resin yield with or without resin-stimulating paste treatment.

Year and tapping	Effect		
	Treat	Family	Treat × Family
**2018**			
1st tapping	**< 0.0001**	0.3882	0.3347
2nd tapping	**< 0.0001**	0.4697	0.9106
3rd tapping	0.0758	0.9698	0.5891
4th tapping^a^	**0.0055**	0.2837	0.5406
5th tapping	**0.0489**	0.1706	0.057
6th tapping	**0.0033**	0.171	0.473
7th tapping	**< 0.0001**	0.9054	0.3687
**2019**			
1st tapping^a^	0.4854	**< 0.0001**	0.4618
2nd tapping	**< 0.0001**	0.0773	0.3321
3rd tapping	**< 0.0001**	0.1107	0.886
4th tapping	**< 0.0001**	0.5306	0.5531
5th tapping	**0.008**	0.3772	0.8548
6th tapping	**0.015**	0.0596	0.3572
7th tapping	**< 0.0001**	0.527	0.9296
8th tapping	**0.0002**	0.8703	0.6549

^a^No resin-stimulating pastes were applied in this tapping.

Significant effects (*P* value < 0.05) are shown in bold.

### Variations among stimulants and tapping rounds

**Figure 1:**
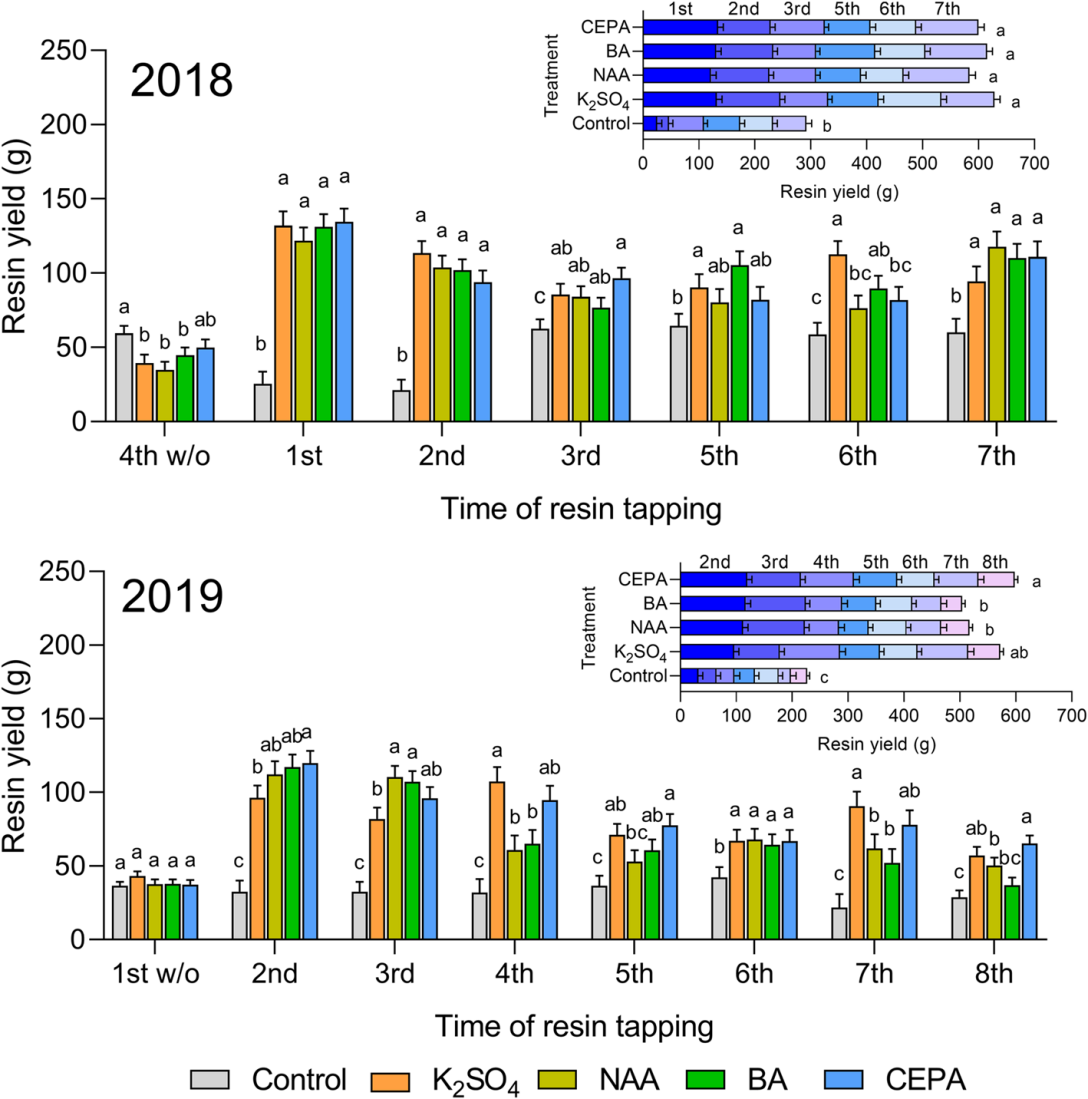
Resin yield of single and total tapping with different resin-stimulating pastes applied. Resin yields of single tapping rounds are shown in the main figures. Resin yields of total tappings are shown in the inset figures. Treatment bars in main figures not sharing a letter are significantly different (Tukey test, P < 0.05). Treatment stacked bars showing total resin production in inset figures not sharing a letter are significantly different (Tukey test, P < 0.05).

The data from the control without stimulants indicated that the response of *P. elliottii* × *P. caribaea* to wounding might be delayed. In 2018, the mean resin yield per tree was only 21.1–25.1 g at the first two tappings and suddenly increased to 59.5–64.5 g at the following five tappings. In 2019, although the measurement was taken during the summer, frequent and abundant rainfall might have hampered resinosis. The mean yield was 32.0–36.5 g at the first five tappings, then increased to 42.4 g at the 6th tapping, and decreased to 21.8–28.9 g at the last two tappings (Fig. 1).

All four stimulants were able to significantly increase resin yield compared with the control, and the effect was most prominent at the beginning in both 2018 and 2019. Approximately 4.5- to 5.4-fold increases in the mean yields were observed by the four stimulants compared to wounding alone at the first and second tapping in 2018, while only 1.2- to 2.0-fold increases were found at the following tappings. In the 4th tapping round, the withdrawal of stimulants resulted in a 25.1–44.5% yield decrease in treatment groups compared to the control group, indicating that the residue effects of the four stimulants were negative. Although the K_2_SO_4_ treatment yielded higher production than the other stimulants at the 6th tapping round, the yield at the other tapping rounds and the sum of the yield were similar among stimulating pastes. Overall, the application of the K_2_SO_4_ treatment was most effective in the first year of resin production.

The second year, we started tapping during a rainy summer, and no trees were treated with stimulants at the first tapping. As expected, no variations were detected among the treatment groups. At the second tapping, which was the first treatment in 2019, approximately 3.0- to 3.7-fold increases upon application of stimulants were detected. Increases between 1.3- and 4.2-fold in resin yield were found at the following six tappings. Variations were detected among different stimulants at most of the tapping rounds, although the overall resin yield in the CEPA treatment was the highest, which was significantly higher than that of the NAA and BA treatment groups but insignificantly higher than that of the K_2_SO_4_ treatment group. Although the K_2_SO_4_ treatment yielded less resin than the other stimulants at the 2nd and 3rd tapping, it gave an at least 50% higher yield than that from the control. When compared to NAA and BA, CEPA was the best treatment for the second-year tapping, and the yield for each tapping was 65.3–119.9 g.

### Correlation between resin yield and growth

**Figure 2:**
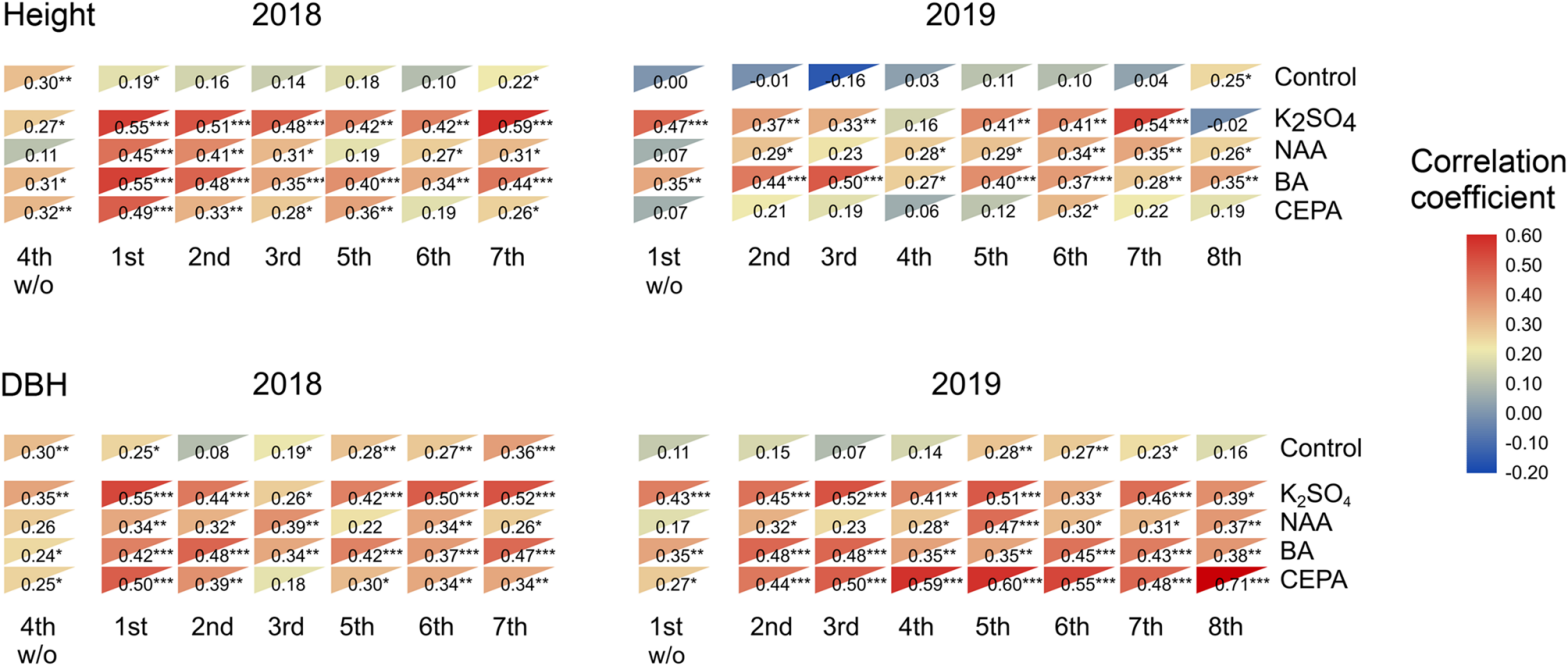
Correlation coefficient between resin yield and height or DBH. The number in the triangle is the correlation coefficient between corresponding resin yield and height or resin yield and DBH. The 4th tapping of 2018 and the 1st of 2019 were performed without (w/o) any resin-stimulating pastes. Asterisks represent the significance of the correlation coefficient. *P < 0.05, **P < 0.01, ***P < 0.001. *DBH* diameter at breast height, *Control* tapping without applying resin-stimulating pastes.

The influence of internal factors, especially tree size on resin yield, are key concerns related to stand management. To test the possibility of using tree size to predict resin yield in the absent or present of stimulants, the correlation coefficients between resin yield and height or diameter at breast height (DBH) were determined (Fig. 2). When no resin stimulating paste was used (e.g., the control group, the 4th tapping round in 2018 and the first tapping round in 2019), DBH showed stronger correlations with resin yield than height. There were 15 correlation coefficients lower than 0.20 between resin yield and height, and there were only 7 correlation coefficients lower than 0.20 between resin yield and DBH. For each tapping round, the correlation coefficients fluctuated without an obvious pattern. When using resin stimulating pastes, the correlation coefficients between resin yield and height or DBH ranged from weak to moderate positive, except for some individual tapping rounds which were non-significant. It was indicated that applying chemical stimulants did not negatively affect the correlation between resin yield and growth, and allow for some degree of predictability in resin yield by tree size.

### Effect of stimulants on resin composition

**Figure 3:**
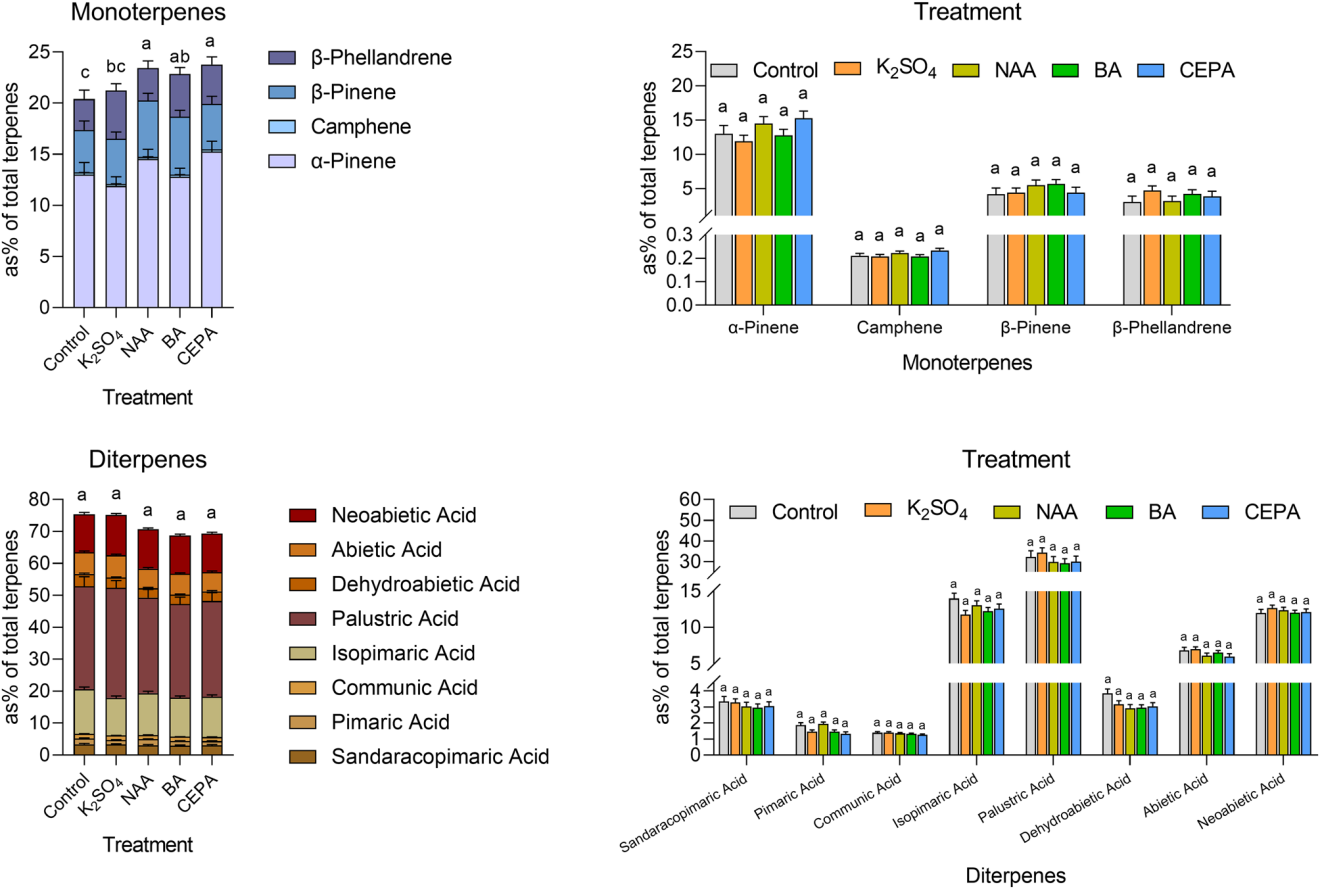
Concentration (as % of total terpenes) of the main resin components from the *P. elliottii* × *P. caribaea* trees with or without resin-stimulating paste application. The estimated marginal means of the percentage concentration of each component are shown in the bar plot. Treatment bars not sharing a letter are significantly different (LSD test, P < 0.05).

Pine resin is mainly composed of a variety of terpene compounds, and the component types and their percentages vary among pine species. The treatment and family effects on resin composition were detected in four of the families (F41, F42, F47 and F48); the measured concentration (as a percentage of total terpenes) of each resin component is shown in Supplementary Table S1. In all samples, the percentage of α-pinene was much higher than that of other monoterpenoids, but the percentages of β-pinene and β-phellandrene were similar. Palustric acid represented the largest fraction of diterpenoids, followed by much lower amounts of isopimaric acid, neoabietic acid and abietic acid. Variance analysis showed that, except for total monoterpenes, the effect of stimulant treatments was nonsignificant on these resin components, which indicated that the application of resin-stimulating pastes minimally affected the proportion of resin composition (Table 2). The total monoterpenes in the NAA, BA and CEPA treatment groups were significantly and slightly higher than those in the control group (Fig. 3). Correspondingly, the total diterpene in these treatment groups was lower than that in the control group, but there was no significant difference.

**Table 2:** Significance levels (*P *values) from the analysis of variance conducted on the main resin components of four *P. elliottii* × *P. caribaea* families with or without resin-stimulating paste treatment.

Resin components	Effect		
	Treat	Family	Treat × Family
**Monoterpenes**	**0.042**	0.056	0.192
α-Pinene	0.193	0.306	0.757
Camphene	0.277	**0.026**	0.061
β-Pinene	0.555	0.119	0.633
β-Phellandrene	0.494	0.873	0.966
**Diterpenes**	0.359	0.623	0.638
Sandaracopimaric acid	0.590	0.065	0.743
Pimaric acid	0.063	**0.010**	0.274
Communic acid	0.333	0.203	0.744
Isopimaric acid	0.204	0.308	0.964
Palustric acid	0.578	0.590	0.390
Dehydroabietic acid	0.276	**0.039**	0.701
Abietic acid	0.266	0.229	0.121
Neoabietic acid	0.711	0.386	0.353

Significant effects (*P* value < 0.05) are shown in bold.

A significant family effect was detected in three components, camphene, pimaric acid and dehydroabietic acid (Table 2), the relative abundances of which were relatively low (Fig. S1). No significant variations among the families were detected in the high abundance components, such as α-pinene or palustric acid.

## Discussion

Due to the much higher growth rate than the other *Pinus* species, the *P. elliottii* × *P. caribaea* hybrid has been developed as one of the main species for afforestation and joint production of timber and resin in southern China. Analyses of external and internal factors influencing resinosis provide opportunities to increase resin yield and tapping efficiency in *P. elliottii* × *P. caribaea* plantations^25^. In the current study, tests were conducted on an experimental stand suffering severely from the impact of Typhoon Hato. The relatively low yields for each tapping were detected in the trees without stimulant application when compared to the yields previously obtained in other stands. The resin yield reduction might be caused by crown and branch damage during typhoons, since trees with larger crown biomasses might produce more resin^20,21^. To evaluate the increasing effects of plant growth regulators and metal ions, four kinds of pastes with different resin-promoting mechanisms were applied in the stand. CEPA can degrade to ethylene, which accumulates in response to wounding and thus increases the resin yield^26,27^, while NAA promotes resin yield by promoting the biosynthesis of ethylene and inducing the differentiation of resin canals^7^. As a synthetic precursor of salicylic acid (SA), BA promotes resin yield by activating the SA-mediated defense response to pathogens^15^. The stimulatory effect of potassium ions may be related to their roles as activators of terpenoid synthases^9^. All these stimulants significantly increased the resin yield in *P. elliottii* × *P. caribaea* when compared to the control. For a single tapping and harvest, the application of resin-stimulant pastes increased the resin yield by up to 5.38 times (Fig. 1). For the total resin yield, the application of resin-stimulant pastes promoted the total yield by up to 2.15 times in 2018 and 2.63 times in 2019. This is more or less in agreement with previous studies of slash pine in Brazil, which found that the application of resin-stimulant pastes in the first year and the second year can increase the total resin yield by approximately 2.14 and 1.66 times, respectively^12^.

In contrast to the common use of resin-stimulant pastes in the Americas, the traditional bark streak tapping method without stimulants has commonly been used in China^28^. The high cost of pastes is one of the concerns, and it is even higher than labor costs in some places. The main component of the commonly used paste is CEPA, which is expensive and toxic. How to reduce the cost of pastes and achieve a similar promoting effect is important for the popularization and application of resin-stimulant pastes. Previous studies indicated that it was possible to replace commercial resin-stimulant pastes containing CEPA with the pastes containing potassium ions in *P. elliottii*^9,16^. Our study showed that there was no significant difference in total resin yield between the K_2_SO_4_ and CEPA treatment groups, while the resin yield of the treatment group with NAA or BA was lower than that of the CEPA treatment group in 2019. In China, the cost of CEPA to prepare 1 L of paste is 3.5 times that of K_2_SO_4_, and the latter is nontoxic. Therefore, resin-stimulant pastes with K_2_SO_4_ as the main component may be more competitive in the market.

Analysis of current data separately collected from each tapping round provided information on resin production dynamics in response to stimulants. It was found that the increases were dramatic during the first two tapping rounds and then fade, implying that the promoting effects might be regulated by resource allocation trade-offs^29^. When wounding and stimulants were applied together, trees first prioritized defense (resin production) over growth. However, if the treatments last longer than 2–3 weeks, resources allocated to growth might increase. This may also explain why the residue effects of the stimulants were negative. With the withdrawal of stimulants, the treated trees with previously more restricted growth would allocate more resources to growth than the control trees^30^.

As usually detected in many *Pinus* species, resin yield is genetically controlled and genetically or phenotypically correlated with tree size. Significant variations among families in resin yield were detected in *P. massoniana*^20,31^, *P. caribaea*^32^, *P. elliottii*^21^ and *P. pinaster*^33^. At the phenotypic level, resin yields were mostly significantly correlated with DBH, while weak correlations or no significant correlations were found with height^20,32^. In our study, the resin yield differed among families only at the first tapping in 2019 without stimulant treatment, but this genetic variation disappeared after stimulant treatments. It was indicated that although resin yield was genetically controlled in *P. elliottii* × *P. caribaea*, the effects of environmental factors on this trait were greater than the genetic factors. Thus, stimulant application in resin yield selection among families may not be necessary. However, our results also indicated that it was possible to predict the ranking of stimulated resin yield of individual trees by DBH. The phenotypic correlations between resin yield and DBH were medium to strong (ranging from 0.44 to 0.71) with CEPA application in 2019. The mechanisms underlying the relationship between tree size and resin flow remain unclear. One possibility is that the fast-growing trees that are often considered to have more and larger resin ducts^34^ allow more resin-synthesized flow to the wounding site.

Whether the application of resin-stimulant pastes will affect the quality of resin is also a topic of concern for resin producers. The variations in the proportion of resin components may affect the processing technology of resin as well as the quality of rosin and turpentine products. Previous studies reported that the applications of chemical stimulants influenced the abundance of some components. It was found that NAA application increased the content of β-pinene in *P. elliottii*^12^, and metal ions such as K^+^, Fe^3+^, and Cu^2+^ increased the percentage of camphene^9^. In our findings, the four monoterpene and eight diterpenoid constituents in resin showed no changes after the application of all the resin-stimulant pastes. However, the overall percentages of monoterpenes slightly increased in the resin of NAA-, BA- or CEPA-treated trees. These data were consistent with previous findings showing that the proportion of monoterpenes to resin acids showed a faster increase in response to wounding and stress than other terpenes^35,36^. In addition, our results showed that the percentages of monoterpenes did not change in K^+^-treated trees compared to the control, which might be the difference between the pastes with or without plant growth regulators. At the family level, we only detected genetic variations in camphene, pimaric acid and dehydroabietic acid. This may have been caused by the relatively small sample size used in the resin composition analysis, which might have made the detection of significant genetic effects difficult.

## Conclusion

Our study is the first to examine the effects of four different types of resin-stimulant pastes (K_2_SO_4_, NAA, BA, CEPA) on resin production in the *P. elliottii* × *P. caribaea* families. Considerable resin-promoting effects were detected for all four chemical stimulants, especially at the beginning of the stimulating treatments. The CEPA- and K_2_SO_4_-based pastes performed better than the other two pastes in both years. When continuous tappings were conducted with the stimulant paste application, their resin-promoting effects dramatically decreased. The internal effects of tree size became more relevant when combined with external chemical stimulants, and faster growth meant higher resin yield in these cases. The application of pastes with plant growth regulators slightly increased the overall proportion of monoterpenes, while the main components of resin were not affected.

In summary, resin-stimulating paste with K_2_SO_4_ as the main component is recommended for *P. elliottii* × *P. caribaea* in South China when the cost of chemicals and the stability of resin compositions are considered. The best resin-promoting effects will be obtained by appropriate interval application and in fast-growing trees.

Our results also clarified that *P. elliottii* × *P. caribaea* has excellent potential for resin production, especially using resin-stimulating pastes. It is feasible to offer both resin and wood in one planting rotation. The present study provides a valuable reference for the comprehensive utilization of this hybrid in other planting areas.

## Materials and methods

### Study site

**Figure 4:**
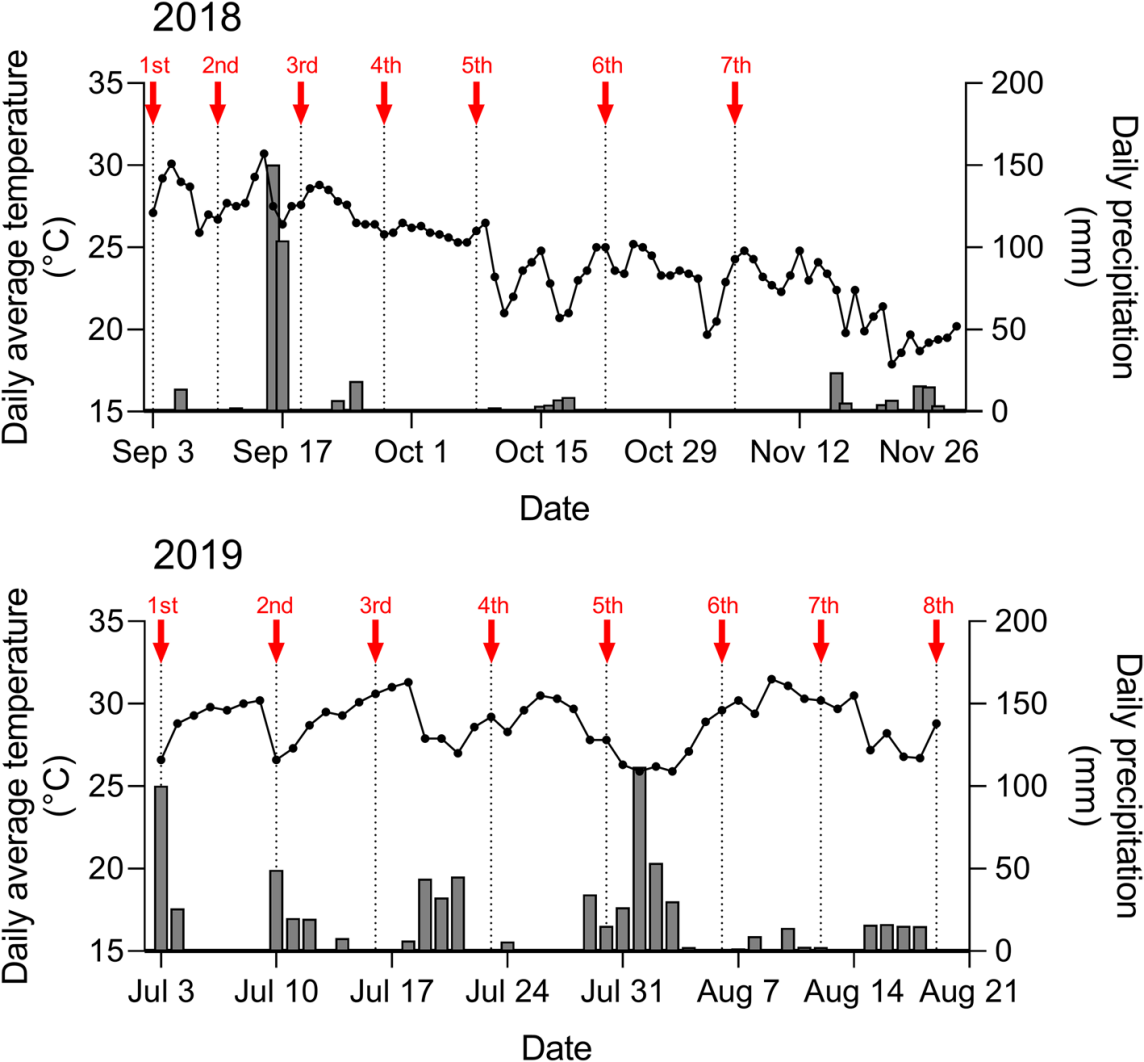
Daily average temperature and daily precipitation in 2018 and 2019 during the resin tapping period. The resin tappings are indicated with red arrows. Data were obtained from the China Meteorological Data Network (http://data.cma.cn) choosing the Taishan meteorological station. The bar plot represents the daily precipitation, and the line graph represents the daily average temperature.

The study was conducted at a *P. elliottii* × *P. caribaea* stand in Taishan Hongling Seed Orchard of Taishan City, Guangdong Province, China (112°49ʹE, 22°11ʹN). The site is located at an elevation of 30 m above sea level and is covered by red soils derived from granites with pH values ranging from 5 to 5.5. The area has a subtropical maritime monsoon climate. The annual mean temperature is approximately 21.8 °C, and the annual precipitation averages 1940 mm. The weather conditions were good for pine growth except for the 13th typhoon Hato in the summer of 2017. After the typhoon, the trees in the experimental stands did not fall down, but almost half of the crown and top branches were destroyed. The current study started 1 year after the typhoon. The daily temperature and precipitation data during the experimental periods of 2 years (2018 and 2019) were obtained from the nearest meteorological station (112°47ʹE, 22°15ʹN, Fig. 4). In 2018, the experiment began in September and lasted for 12 weeks, the daily average temperature gradually decreased (from nearly 30 °C to 20 °C), and daily precipitation was also reduced except in September. In 2019, the experiment began in July and lasted for 7 weeks, and the daily average temperature fluctuated significantly, but the overall temperature remained between nearly 26 °C and 32 °C. There was a large quantity of precipitation accompanied by cooling. Clearly, the characteristics of the daily average temperature and precipitation in the two experiments in the 2 years examined were different.

### Treatment and resin tapping

The study plots were established in a *P. elliottii* × *P. caribaea* progeny test stand at a spacing of 3 m × 3 m, which was planted in June 1999. The hybrids were produced through partial factorial mating of 6 parents of *P. elliottii* and *P. caribaea* var. *hondurensis*, and 33 F_1_ families were planted. The experimental layout was a split-plot design with four treatments and one control acting as whole plots and the 33 F_1_ families randomly assigned to single-tree subplots. The experiment was replicated in six blocks. The study materials consisted of 990 individual trees. The average height of these trees was 24.82 m, and the average DBH was 23.52 cm.

A downward-pointing V-shaped groove reaching the secondary xylem was cut every week. The groove reached approximately one-fifth of the tree circumference. The tapping was carried out seven times in 2018 and eight times in 2019, the frequency of which is shown in Fig. 4. The fourth tapping in 2018 and the first tapping in 2019 were performed without resin-stimulant paste and were used as the internal controls. Four kinds of resin-stimulant paste were used in this study: K_2_SO_4_ paste (with 500 mM K_2_SO_4_), NAA paste (with 1 mM NAA), BA paste (with 10 mM BA) and CEPA paste (with 3% Ethrel). Each paste was supplemented with 20% sulfuric acid as the basal active component and 40% rice husk powder as a thickening agent to increase the residence time on the wound^13^. Approximately 12 mL of the pastes was evenly applied to the top portion of the fresh wounds.

### Tree height, DBH and resin yield measurement

The height of each tree was determined by a Haglöf Vertex IV ultrasonic (Haglöf Sweden AB, Långsele, Sweden) clinometer, and the DBH of each tree was measured by a breast diameter ruler. The resin yield of each tree was collected and weighed after each tapping by an electronic balance.

### Chemical composition analysis of resin by GC–MS

The resin samples were collected from four of the families (F41, F42, F47 and F48) and three technical replicates were used. The freshly collected resin was treated and analyzed as previously described^37^. Briefly, 0.2 g of resin was dissolved in 2 mL of ethyl alcohol containing 50 µL tetramethylammonium hydroxide. Gas chromatography–mass spectrometry (GC–MS) was performed by Agilent 7890B GC and 5977A MS for qualitative and quantitative analyses of resin components. For GC, the initial temperature of the program was 60 °C for 2 min, and the temperature rose to 260 °C for 10 min at a rate of 4 °C/min. The temperature of the vaporization chamber and detector was maintained at 260 °C. Component detection was conducted by a hydrogen flame ionization detector (FID) with high purity nitrogen (0.1 MPa) as the carrier gas. The hydrogen flow rate was 50 mL/min with an air flow of 50 mL/min. The split ratio was set to 1:100, and the sample injection volume was 1.0 µL. The mass spectra were compared against the NIST and in-house database to identify the components. Each component content was normalized to the total peak area of the components.

### Statistical analyses

Statistical analysis was performed using SAS version 9.4 (SAS Institute, Cary, NC, USA) and the MIXED procedure for all variables. The effect of clonal genotypes and treatments on the growth and physiological parameters were analyzed using the following mixed model:

$$ Y_{jkl} = \mu + T_{j} + G_{k} + B_{l} + TG_{jk} + TB_{jl} + \varepsilon . $$

where *Y*_*jkl*_ is the corresponding variable (height, DBH, resin yield); μ is the overall mean; *T*_*j*_, *G*_*k*_ and *B*_*l*_ are the effects of jth stimulant treatment, kth family, and lth block, respectively; *TG*_*jk*_ and *TB*_*jl*_ are the corresponding interactions effects; and ε is the experimental random error. The model included the random effect of block and the interaction between stimulant treatment and block (*TB*_*jl*_), as well as the other fixed effects. Multiple comparison tests were conducted for the differences of the least-squares means using Fisher’s LSD test. Pearson’s correlation was employed to determine the correlation coefficient between two indicators. When the probability *P *value is less than 0.05, the F test reaches a significant level. The correlation heatmaps were constructed by using the Java-based tool TBtools 1.0^38^. A general linear model (GLM) was used for each resin component, total monoterpenes and total diterpenes analysis within four families and five treatments (one control group included).

### Plant collection and experiments statement

The *P. elliottii* × *P. caribaea* families were bred by Guangdong Academy of Forestry and Taishan Hongling Seed Orchard. All the plant experiments were in compliance with relevant institutional, national, and international guidelines and legislation.

## Supplementary Information

Supplementary Figure S1.

Supplementary Table S1.

## Data Availability

The datasets used or analyzed during the current study are available from the corresponding author on reasonable request.
