# Supplementary material for: Efficient resin production using stimulant pastes in Pinus elliottii × P. caribaea families
Source: Sci Rep. 2022 Jul 30;12:13129. doi: 10.1038/s41598-022-17329-2 (PMC9338930; doi:10.1038/s41598-022-17329-2)
Supplement: Supplementary file 1 — Supplementary Figure S1. [file 41598_2022_17329_MOESM1_ESM.pdf]

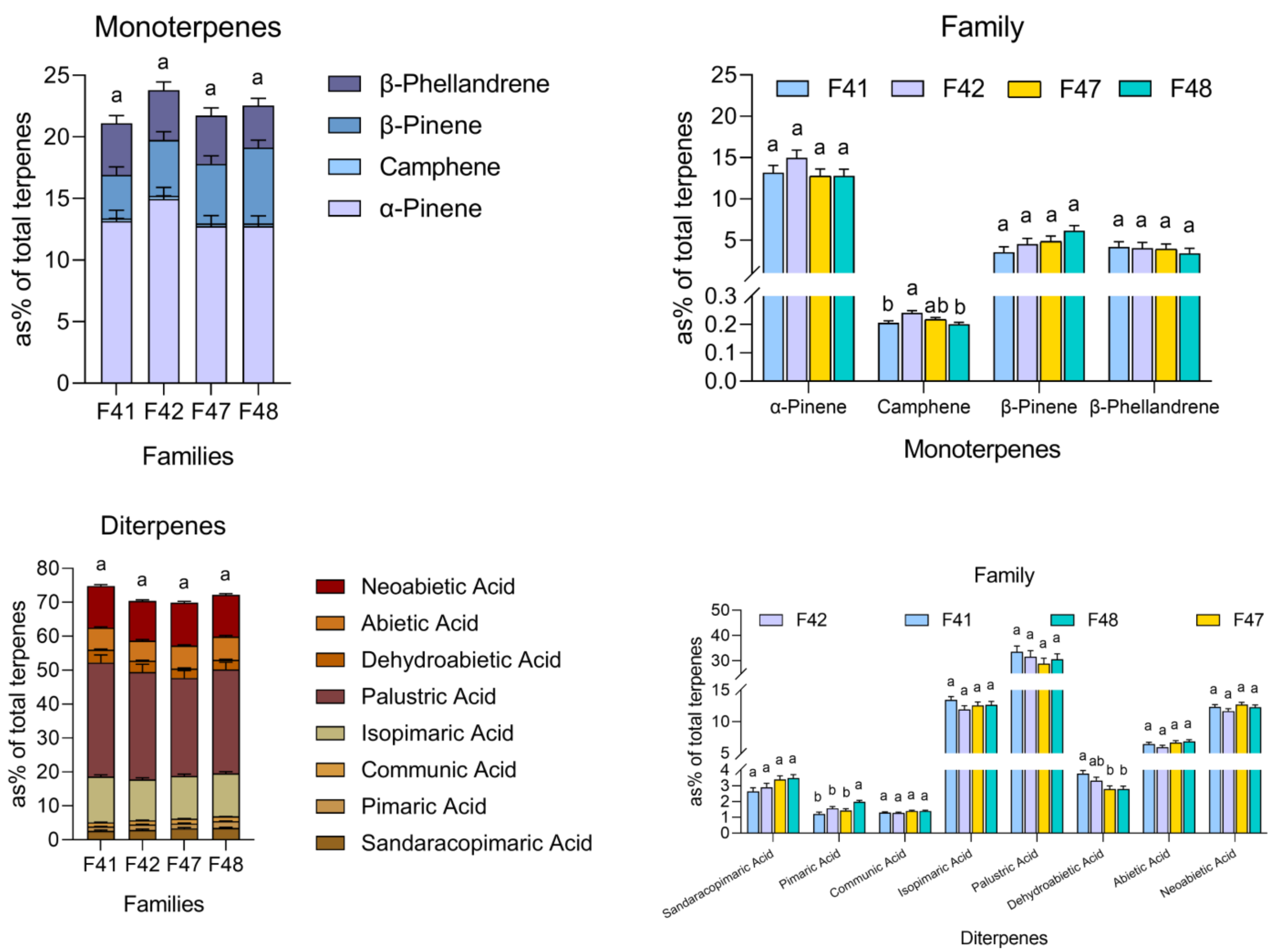

Fig. S1 Concentration (as% of total terpenes) of the main resin components from the *P. elliottii* × *P. caribaea* trees in four elite families.

Estimated marginal means of the percentage concentration of each components were showed in the bar plot. The bar of the treatment with different lowercase letter indicates the significant difference (LSD test, P<0.05).
